# Relationship of body mass index to percent body fat determined by deuterium isotopic dilution and impedancemetry among Tunisian schoolchildren

**DOI:** 10.4314/ahs.v23i3.77

**Published:** 2023-09

**Authors:** Ben Jemaa Houda, Khlifi Sarra, Inchirah Karmous, Jamoussi Henda, El Kari Khalid, Aguenaou Hassan, Aouidet Abdallah, Mankaï Amani

**Affiliations:** 1 Nutrition Department, Higher School of Health Sciences and Technics, University of Tunis El Manar, Tunis, Tunisia; 2 Laboratory SURVEN, National Institute of Nutrition and Food Technology of Tunis, Tunisia; 3 Research Unit “Obesity: Etiopathology and Treatment, UR18ES01, National Institute of Nutrition and Food Technology, Tunis, Tunisia; 4 Joint Unit of Nutrition and Food Research (URAC39), CNESTEN-Ibn Tofaïl University, Morocco

**Keywords:** BMI, body fat, deuterium isotopic dilution, impedancemetry, obesity

## Abstract

**Objectives:**

This study aims to evaluate the relation of body mass index (BMI) to fat mass among children by two techniques impedancemetry and deuterium oxide dilution (D_2_O).

**Methods:**

This study was carried out in 156 schoolchildren aged between 8 and 11 years. The children received interrogation specifying lifestyle and food habits. Body composition was determined using the impedancemetry and D2O technique.

**Results:**

The results showed a difference between the percentage of obese and overweight children according to BMIZ classification (30.1%), bioelectrical impedance method (14.7%) and D_2_O technique (42.9%). Despite the difference between the last two classifications, we found a significant correlation between body fat percentages determined by impedancemetry and D_2_O technique (r = 0.695, p<0.01). Bioelectrical impedance analysis underestimated %BF by 78.02% in overall children, by 70.05% in boys and by 84.73% in girls compared to D_2_O technique.

**Conclusion:**

This study has demonstrated that the percentage of overweight and obesity varied according the methods used. Further development of body composition methods is needed in children for the real determination of the obesity prevalence and therefore a better monitoring of this public health problem.

## Introduction

Obesity is a major public health problem due to its association with serious chronic diseases such as type 2 diabetes, cardiovascular disease, and many cancers.^1^ In recent decades, the prevalence of obesity in children has increased worldwide. More than 330 million children and adolescents aged 5–19 years were overweight or obese in 2016.^2^

In order to limit this burden, a high priority should be given to screening and monitoring the nutritional status of children. Monitoring the prevalence of obesity to plan services for the provision of care and to assess the impact of policy initiatives is essential.

Body mass index for age z-score (BMIZ) is the pragmatic measure to assess children's obesity clinically and BMI charts are widely used for counselling families about children's weight management over time.^3^ Obviously, even if these measures provide useful information, other methods are indicated to provide accurate information on body composition.^4^ There is increasing interest in the assessment of body composition in children, for several reasons.^5^ Body composition measurements could inform clinical diagnosis, improve routine management and help determine nutritional requirements. Various established methods are used for body composition assessment, including Bioelectrical impedance analysis (BIA), dual-energy x-ray absorptiometry (DXA) and deuterium oxide dilution (D_2_O) technique.^6^

It has been evident in several studies that the use of BIA, an easy handling and cheap method is practical in assessing of the body composition.^7^ These techniques are based on the frequency-dependent response of different body tissues to the application of a low alternating current. However, these methods require specific prediction equations that are population specific. These equations having been developed based on reference data in a particular population and thus are often inadequately adapted to specific subjects being studied, leading to significant error.^8^

Deuterium oxide (D_2_O) is a reference technique for measuring body composition, and is valid for all groups. D_2_O concentrations serves as a marker for total body water from which fat free mass and fat body mass (BF) are derived.^9^, ^10^

The objectives of this study are; First to determine the body composition of schoolchildren aged between 8 and 11 years. Then, to compare the overweight and obesity frequency determined by BMIZ, Tanita system and deuterium technique. Finally, to study the correlation between socio-economic status, dietary habits and nutritional status determined by the reference technique.

## Materials and Methods

### Study participant

The study was conducted in urban region during 2018–2019. A total of 156 children (80 boys and 76 girls) aged between 8 and 11 years were recruited from three randomly selected primary schools. The sample selection was conducted randomly on two levels, schools and children. Inclusion criteria comprised children with a good general health and an absence of an acute illness that could cause abnormalities in body composition. Written consent was obtained from parents.

### Data collection

A structured questionnaire was completed by the investigator. Students were interviewed at school. Information obtained included student's identification and student's anthropometric measurements taken.

### Anthropometric measurements

Anthropometric measurements were performed on children using standardized procedures. Body weight was measured using a digital electronic scale (Seca, Hamburg, Germany, 896 (150±0.1kg) with minimal clothing and no shoes. Height was measured to the nearest 0.1 cm using a mobile vertical anthropometer (ALTUREXATA®). BMI was calculated as weight in kilogram divided by the square of height in meter (Kg/m^2^). BMIZ and Height-for-age z-scores (HAZ), and were calculated using WHO growth standard reference for children and youth 5–19 years of age.^11^ BMIZ was used to categorize children as normal weight (BMIZ< + 1) and overweight/obese (BMIZ> + 1) groups. Waist circumference (WC) was measured to the nearest 0.1 cm with a two-meter long, flexible and inelastic anthropometric tape at the midpoint of the upper arm and with the arm hanging straight at the side of the body. All the measures were performed twice.

### Deuterium oxide dilution

In our study, the percentage of body fat (%BF) was determined by an isotope dilution technique using the deuterium oxide according to the protocol which was previously described by Ben Jemaa et al., 2019. 12 Participants orally consumed a dose equal to 0.5 g/kg of body weight of deuterium oxide 99.8%. Predose deuterium abundance was obtained from one fasted saliva sample (approximately 1 mL) collected from the children through chewing a ball of cotton wool, which was then squeezed into a syringe to extract the saliva. The children were instructed to refrain from any food or fluid for at least 30 min before the post-dose saliva samples. All samples were stored at −20 °C and the analysis was performed with by a Fourier transform infrared spectrophotometer (FTIR Agilent 4500, Malaysia). The calculation of the body composition was performed following the manufacturer's instructions.^9^ Based on the fundamental principle of dilution, concentration and volume of deuterium present and measured in saliva are correlated before and after dose ingestion. This principle disclosing the total body water (TBW) volume provides a calculation of free fat mass (FFM), using the specific hydration coefficients.^13^ The absolute Fat Mass (FM) was derived by subtracting FFM from body weight, based on the two-compartment body composition model and %BF was then calculated.

### Impedance analysis

The impedance measurements were performed by the scale TANITA (TBF-401A, Tokyo, Japan). It is presented as a pedestal analyser and measuring is done in minutes. Data were measured according to the manufacturer's guidelines. Each participant must be standing in light clothing and bare feet on the metal imprints shaped soles which constitute the electrodes. Information on %BF, FM, FFM and TBW are provided. For interpreting the body fat percentages provided by both methods (electrical impedance and isotope dilution) of the children surveyed, we passed thresholds to define excess body fat: The excess fat mass is defined in girls by %BF≥ 30% and %BF≥ 25% in boys.^14^

### Statistical Analysis

Data analysis was performed using SPSS Version.21. Continuous variables are presented as mean ± standard deviation, and categorical variables are presented as relative frequencies. For the study of correlations between the quantitative parameters, we used Pearson correlation coefficients. Chi-square and Fisher's exact test were used in comparing frequencies between two independent samples.

Bland Altman analysis was used to examine the agreement between impedance analysis and deuterium dilution in measuring total body fat.^15^

## Results

Anthropometric parameters for 156 children (80 boys and 76 girls) are listed in table 1. Mean age was 9.30±0.97 years with mean BMIZ of 0.28±1.17, and mean HAZ of 0.37±0.89. Any significant difference between genders was found for weight, height, waist circumference and BMIZ.

**Table 1:** Anthropometric characteristics of children

Characteristics	All (n=156) Mean ±SD	Boys (n=80) Mean ±SD	Girls (n=76) Mean ±SD	p
**Age (year)**	9.30±0.97	9.25±0.95	9.35±1.00	0.522
**Weignt (kg)**	32.68±7.08	31.89±6.49	33.54±7.61	0.146
**Height (cm)**	137.38±8.37	136.61±7.98	138.20±8.74	0.237
**BMI (kg/m^2^)**	17.18±2.66	16.93±2.21	17.44±3.06	0.233
**BMIZ**	0.28±1.17	0.27±1.09	0.30±1.24	>0.999
**HAZ**	0.37±0.89	0.30±0.82	0.44±0.97	0.445
**Waist circumference (cm)**	60.31±6.41	59.56±5.59	61.09±7.13	0.137

BMIZ: Body Mass Index z-score; HAZ: Height-for-age z-scores

Percentage of body fat determined by the Tanita system were significantly lower than those found by D_2_O technique (21.06±7.74 vs 26.99±6.96, p<0.0001) in all children (Table 2). The same result was found in boys and in girls (16.85±5.38 vs 24.06±5.52, p<0.0001 and 25.49±7.39 vs 30.08±5.68, p<0.0001 respectively). Tanita system underestimated %BF by 78.02% in overall children, by 70.05% in boys and by 84.73% in girls compared to D_2_O technique.

**Table 2:** Comparison between Tanita System and deuterium oxide dilution method in measuring percentage of body fat in children

	%BF Tanita System	%BF D_2_O Technique	P	%BF Tanita System/%BF D_2_O Technique
**Overall (n=156)**	21.06±7.74	26.99±6.96	<0.0001	78.02%
**Boys (n=80)**	16.85±5.38^a^	24.06±5.52^b^	<0.0001	70.05%
**Girls (n=76)**	25.49±7.39^a^	30.08±5.68^b^	<0.0001	84.73%

Comparison between girls and boys for Tanita System; **a:** (p<0.0001)

Comparison between girls and boys for D_2_O technique: **b:** (p<0.0001)

The weight status results based on BMIZ, %BF determined by Tanita System and D2O technique are shown in Table 3. The frequency of obese and overweight subjects determined by BMIZ, Tanita System and deuterium technique was 30.1%, 14.7% and 42.9% respectively with a statistically significant difference. When the sample was stratified by gender, results of the BMIZ revealed that 27.5% of boys and 32.9% of girls were classed as overweight or obese. According the Tanita system, 7.5% of boys and 22.4% of girls were overweight or obese. Using the deuterium dilution technique as the reference method, Overweight and obesity affected 36.3% of boys and 50% of girls. Statistical analyses indicated that only the percentage of overweight determined by Tanita system differed significantly as for as gender is concerned (7.5% in boys vs 22.4% in girls, p <0.01).

**Table 3:** Distribution of children's corpulence according BMIZ, % body fat determined by Tanita System and % body fat determined by the deuterium oxide dilution method

		BMIZ	Tanita System	D_2_O Technique	p
**Overall** **(n=156)**	Normal	69.9 %	85.3 %	57.1 %	<0.001
	weight	(109/156)	(133/156)	(89/156)	
	Overweight	30.1 %	14.7 %	42.9 %	
		(47/156)	(23/156)	(67/156)	
**Boys** **(n=80)**	Normal	72.5%	92.5%	63.7%	<0.001
	weight	(58/80)	(74/80)	(51/80)	
	Overweight	27.5%	7.5%	36.3%	
		(22/80)	(6/80) *	(29/80)	
**Girls** **(n=76)**	Normal	67.1%	77.6%	50%	0.001
	weight	(51/76)	(59/76)	(38/76)	
	Overweight	32.9%	22.4%	50%	
		(25/76)	(17/76) *	(38/76)	

D_2_O: deuterium dilution technique

* significant difference between the gender (p <0.01)

**Figure 1:**
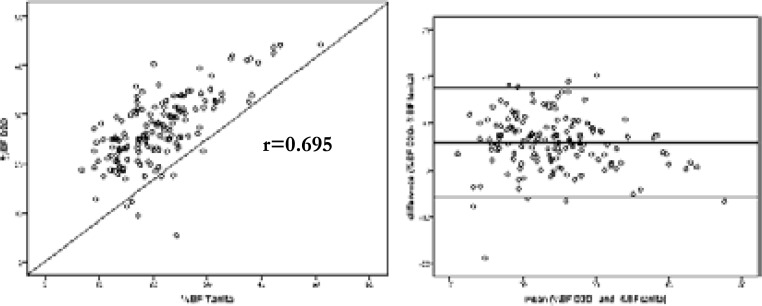
Correlation plot between body fat percentage determined impedance analysis (%BF Tanita) and by the deuterium oxide dilution method (%BF D2O) (a) and by the Bland-Altman plot (b)

BMIZ was significantly and positively correlated with %BF determined by the Tanita system (r=0.628, p<0.01) and with %BF determined by D2O (r=0.542, p<0.01). A significant correlation between %BF values determined by the Tanita system and those reported by de D_2_O method was revealed (r=0.695, p<0.01) (Figure 1a). The Bland-Altman plot displays the difference in %BF obtained from D2O technique and the Tanita system plotted against the mean of %BF of both measures (Figure 1b). The SD of difference was 5.84%, the bias was 5.93, and the limits of agreement (mean difference±1.96 SD) between the two methods were 5.61 to 17.39. This interval is wide, reflecting the great variation of the differences. They show that even on the most optimistic interpretation, there can be considerable discrepancies between the two methods and that the degree of agreement is not acceptable.

Relationship between Waist circumference and the percentage of body fat was illustrated in Table 4. A significant correlation was observed between WC and %BF in both genders of the study sample (boys, r = 0.33; girls, r = 0.55; (p< 0.05)) and in overweight/obese children (boys r = 0.41, (p < 0.05); girls, r = 0.54 (p = 0.001)). However, there was no significant correlation between %BF and WC in normal weight boys and girls. A significant correlation was observed between WC and FM in normal weight (boys: r= 0.42; p = 0,02; girls: r = 0.42; p = 0,008) and also in overweight/obese in both genders (boys: r = 0.71; p < 10^-3^; girls = 0.74; p < 10^-3^).

**Table 4:** Pearson's correlation coefficients (***r***) of waist circumference with each of fat mass and percentage of body fat

		WC	
		%BF	FM
**Boys**	Total (n = 80)	0.33; p = 0.003	0.6; p < 10^-3^
	Normal weight (n = 51)	NS	0.42; p = 0.02
	Overweight (n = 29)	0.41; p = 0.03	0.71; p < 10^-3^
**Girls**	Total (n = 76)	0.55; p < 10^-3^	0.77; p < 10^-3^
	Normal weight (n = 38)	NS	0.42; p = 0,008
	Overweight (n =38)	0.54; p = 0.001	0.74; p < 10^-3^

## Discussion

In this study, %BF determined by D_2_O technique were significantly higher than those found by Tanita. The same result was found in the two genders. So compared to D_2_O technique, Tanita system underestimated %BF. The same results were found in the Lebanon study^16^ and Bangladesh study,^17^ done on 66 Bangladeshi children aged 4-10 Years. However, a previous study has reported that BIA methods overestimate %BF in lean subjects.^18^ This difference between the studies could be explained by the type of impedance meter used and characteristics of the studied population.

Our results illustrated a correlation between the %BF obtained by Tanita system and BMIZ has been demonstrated. These results are in agreements with previous study.^19^ Furthermore, a high correlation was found between the %BF obtained by Tanita system and that obtained by D2O technique. The same result was found in a Brazilian study that involved 40 obese teenagers aged from 10 to 19 years.^20^ Camarneiro et al. (2013) evaluated the relationship between body composition determined by bioelectrical impedance and deuterium oxide and anthropometric measures in adolescents and they reported a significant correlation between, these methods.^21^

The classification of the weight state according to the D_2_O technique indicated a rate of excess in fat mass of the order of 42.9%. This is not concordant with the rate of obesity and overweight determined by Tanita system (14.7%) and the BMIZ classification (30.1%). The values found by D_2_O technique are close to those determined by BMIZ classification. This finding concord with other study that report that the increased BMI z-score in childhood is associated with higher percentages of body fat.^22^

The percentage of overweight/obesity determined by the D_2_O technique is significantly higher than those determined by the BIA. This discrepancy between the two methods was reported by previous study, that demonstrate the inaccurate estimation of the Tanita in- built prediction equation compared to deuterium oxide dilution.^17^ In fact, BIA method need to be used in connection with prediction equations derived from a reference standard and may be valid only for a population of similar age, sex, ethnicity, and health status.^23^

Most of the existing BIA prediction equations have been derived from Caucasians. The models used to evaluate obesity require revision before they can be applied in other population because ethnicity influences the body composition.^24^

In this study, overweight/obesity was higher among the girls compared to boys; however, this relationship was statistically significant only for the Tanita results. The effect of gender on overweight and obesity in children remains inconclusive, whereas, the global prevalence of obesity is higher in women than in men.^25^ Changes in body composition occur during puberty in both sexes. In this period there is a rapid increase in body fat, although this increase is slower in males, being overcome by lean body mass.^26^

WC is universally used to define body fatness,^27^ and numerous studies have addressed its capacity to indicate central fat accumulation in children. In the present study, the relationship between WC and each of FM and %BF was explored. Overall, WC was found to be correlated with %BF and FM in both genders. Our results are similar to those of previous studies which report that WC was significantly correlated with Fand with %BF in boys and girls.^28^ Alves Junior et al. reported that the WC is positively correlated with BMI and total fat.^29^ In our study, the correlations were stronger in girls compared to those observed in boys. These results agree with other study which showed that the relationship between WC and body fat is influenced by gender.^28^ This finding was explained previously by the fact that girls have a higher FM than boys and WC may not reflect total fat.^30^

## Conclusion

This study has demonstrated that the frequency of obese and overweight subjects determined by BMIZ, bioimpedance and deuterium technique was statistically different. Bioimpedance underestimate body fat in Tunisian children aged between 8-11 years compared to D_2_O technique. Body composition measurements are important for assessing nutritional status and monitoring clinical outcomes in children. Further development of body composition methods is vitally needed in children.
